# Does Circulating Antibody Play a Role in the Protection of Piglets against Porcine Epidemic Diarrhea Virus?

**DOI:** 10.1371/journal.pone.0153041

**Published:** 2016-04-06

**Authors:** Korakrit Poonsuk, Luis Gabriel Giménez-Lirola, Jianqiang Zhang, Paolo Arruda, Qi Chen, Lucas Correa da Silva Carrion, Ronaldo Magtoto, Pablo Pineyro, Luciana Sarmento, Chong Wang, Yaxuan Sun, Darin Madson, John Johnson, Kyoung-Jin Yoon, Jeffrey Zimmerman, Rodger Main

**Affiliations:** 1 Department of Veterinary Diagnostic and Production Animal Medicine, College of Veterinary Medicine, Iowa State University, Ames, Iowa, 50011, United States of America; 2 Department of Statistics, College of Liberal Arts and Sciences, Iowa State University, Ames, Iowa, 50011, United States of America; Sun Yat-sen University, CHINA

## Abstract

The contribution of circulating antibody to the protection of naïve piglets against porcine epidemic diarrhea virus (PEDV) was evaluated using a passive antibody transfer model. Piglets (n = 62) derived from 6 sows were assigned to one of 6 different treatments using a randomized block design which provided for allocation of all treatments to all sows' litters. Each treatment was designed to achieve a different level of circulating anti-PEDV antibody via intraperitoneally administration of concentrated serum antibody. Piglets were orally inoculated with PEDV (USA/IN/2013/19338E, 1 x 10^3^ TCID_50_ per piglet) 24 hours later and then monitored for 14 days. Piglets remained with their dam throughout the experiment. Sow milk samples, piglet fecal samples, and data on piglet clinical signs, body weight, and body temperature were collected daily. Fecal samples were tested by PEDV real-time reverse transcriptase PCR. Serum, colostrum, and milk were tested for PEDV IgG, IgA, and virus-neutralizing antibody. The data were evaluated for the effects of systemic PEDV antibody levels on growth, body temperature, fecal shedding, survival, and antibody response. The analysis showed that circulating antibody partially ameliorated the effect of PEDV infection. Specifically, antibody-positive groups returned to normal body temperature faster and demonstrated a higher rate of survivability than piglets without PEDV antibody. When combined with previous literature on PEDV, it can be concluded that both systemic antibodies and maternal secretory IgA in milk contribute to the protection of the neonatal pig against PEDV infections. Overall, the results of this experiment suggested that passively administered circulating antibodies contributed to the protection of neonatal piglets against PEDV infection.

## Introduction

The Coronaviridae is a large and complex family of enveloped, single-stranded, positive-sense RNA viruses that cause enteric and respiratory disease in humans and animals. Recently-emerged coronaviruses include the severe acute respiratory syndrome (SARS) virus that caused outbreaks of respiratory disease in humans in 2002–2003 and the Middle East respiratory syndrome (MERS) virus identified in 2012 [1]. Contemporary work suggests that bat and bird species are the natural reservoirs of coronaviruses [2].

Five coronaviruses are recognized in swine: three alphacoronaviruses (transmissible gastroenteritis virus (TGEV), porcine respiratory coronavirus (PRCV), and porcine epidemic diarrhea virus (PEDV)), one betacoronavirus (porcine hemagglutinating encephalomyelitis virus (PHEV)), and one species of porcine deltacoronavirus (PDCoV) [3–8]. PEDV, TGEV and PDCoV primarily cause enteric infections in pigs. PRCV is the result of deletion and mutation of the spike gene of TGEV. This virus has a predilection for the respiratory tract, but also has the capacity to produce enteric disease [9]. In contrast, PHEV infection ("vomiting and wasting disease") produces encephalomyelitis, rather than enteritis, and thus is not often considered when differentiating enteric infections [6].

Among the porcine coronaviruses, PEDV has received considerable attention because recently emerged highly virulent strains have caused significant morbidity and mortality in neonatal pigs [10]. Catastrophic outbreaks of PEDV were reported in Korea (1997), China (2005), and Thailand (2007) [11]. Following its detection in the U.S. in April 2013 [12], PEDV is estimated to have caused the deaths of 8 million piglets and economic losses of $481 to $929 million (USD) in 2014 [13].

The primary site of PEDV replication is the cytoplasm of villous enterocytes throughout the small intestine. Infection causes epithelial cell degeneration and villous atrophy, which leads to diarrhea, dehydration, and prolonged shedding of PEDV in feces [14–15]. PEDV viremia has also been reported during the acute stage of infection in young pigs [14, 16–18]. The most common clinical consequence of PEDV infection is diarrhea, i.e. watery and flocculent feces, often accompanied by vomiting [19]. Morbidity and mortality is highly age-dependent, with neonatal pigs the most severely affected. Thus, an outbreak in a naïve swine population may result in 90% mortality in piglets ≤ 2 weeks of age and ≤ 40% mortality in 2- to 4-week-old pigs [12]. This age-dependent variation in mortality is likely the result of slower villus-epithelial repopulation and less developed immune systems in neonatal pigs [15–16, 20–22]. Experimentally-infected 3-week-old pigs showed a significant reduction in average daily gain during the first week post-inoculation and no compensatory weight gain in the following 4 weeks [15]. In the field, Olanratmanee et al. (2010) reported that PEDV infection in pregnant gilts and sows may also have contributed to reduced reproductive performance, including a 12.6% decrease in farrowing rate, a 5.7% increase in the return rate, a 1.3% increase in the abortion rate, and a 2.0% increase in the number of mummified fetuses per litter [23].

It is generally accepted that lactogenic immunity, i.e., anti-PEDV secretory IgA in milk, is central to limiting the replication of PEDV in the intestinal tract and protecting piglets against clinical disease [24]. This concept is primarily derived from research showing that sows with higher anti-TGEV SIgA levels in milk were better able to protect their piglets against clinical TGE [19, 25–27]. These observations are the foundation upon which successful TGEV prevention and control strategies have been based for over 50 years [28]. However, dissimilarities between immunity to PEDV versus immunity to TGEV have not been closely examined and deserve investigation. The question addressed in this project was the effect of colostral (passive) antibody on the protection of neonates against PEDV. Specifically, the objective of this experiment was to quantify the impact of circulating anti-PEDV antibody on the course of PEDV replication and clinical disease in the neonatal pig using a passive transfer model.

## Materials and Methods

### Experimental design

The study was conducted under the approval of the Iowa State University Office for Responsible Research (ISU #2-14-7736-S). Piglets (n = 62) from 6 PEDV indirect fluorescent antibody (IFA)-negative sows were intraperitoneally (IP) administered concentrated serum antibody sufficient to achieve one of 6 targeted levels of circulating anti-PEDV antibody. All piglets were inoculated with PEDV 24 h later and were then observed daily until day post inoculation (DPI) 14 or until humane euthanasia was necessary. Each day, sow milk and piglet fecal samples and data on piglet clinical signs, body weight, and body temperature were collected. Serum samples were collected from sows at DPIs -7 and 14 and from piglets at DPIs -1, 0, and 14 or at the time of humane euthanasia. Fecal samples were tested by PEDV real-time reverse transcriptase PCR (rRT-PCR). Serum, colostrum, and milk were tested for PEDV IgG, IgA, and virus-neutralizing antibody. The data were evaluated for the effects of systemic PEDV antibody levels on the outcomes measured.

### Porcine epidemic diarrhea virus (PEDV) inoculum

The PEDV isolate used in the study (USA/IN/2013/19338E), was isolated in 2013 at the Iowa State University Veterinary Diagnostic Laboratory from piglet small intestine submitted from an Indiana swine farm [10]. This isolate is a highly virulent PEDV strain with >99% genetically homology to Chinese strains reported in 2011–2013 [10]. The infectious dose of the virus in 5-day-old piglets was reported as 0.056 TCID_50_. Under experimental conditions, inoculation of 5-day-old piglets caused watery diarrhea at 1 DPI, virus shedding in feces at 4 DPI and villous atrophy within 4 DPI [50] The inoculum used in this study was the 7th passage of the virus on cell culture.

For use in this study, the virus was serially propagated on Vero cells (African green monkey kidney) in flasks using methods described elsewhere [10, 29]. In brief, Vero cells (ATCC^®^ CCL-81^™^, American type culture collection, Manassas, VA) were cultured in 25 cm^2^ flasks (Corning^®^, Corning, NY) using maintenance medium (minimum essential medium (MEM) (Life Technologies, Carlsbad, CA) supplemented with 10% fetal bovine serum (Life Technologies), 2 mM L-glutamine (Sigma-Aldrich, St. Louis, MO), 0.05 mg per ml gentamicin (Life Technologies), 10 units/ml penicillin (Life Technologies), 10 μg per ml streptomycin (Sigma-Aldrich) and 0.25 μg/ml amphotericin (Sigma-Aldrich). Maintenance medium was decanted from contiguous cell monolayers, the monolayer was washed twice with maintenance medium, and then the flask was inoculated with 0.5 ml of a mixture of PEDV and post-inoculation medium (MEM supplemented with tryptose phosphate broth (0.3%) (Sigma-Aldrich), yeast extract (0.02%) (Sigma-Aldrich) and Trypsin 250 (5 μg/ml) (Sigma-Aldrich)). Flasks were then incubated at 37°C with 5% CO_2_ for 2 h to allow virus adsorption after which 5 ml of post-inoculation medium was added to each flask without removing viral inoculum. Flasks were incubated at 37°C with 5% CO_2_ until cytopathic effect (CPE) was observed and then subjected to one freeze-thaw cycle (-80°C). The contents were harvested, centrifuged at 3,000 x g for 10 min at 4°C to remove cell debris, aliquoted into 2.0 ml microcentrifuge tubes, and stored in -80°C until used.

PEDV titration was performed on confluent Vero cells monolayers grown in 96-well plates (CoStar^™^, Corning^®^). Eight 10-fold dilutions of virus stock solution were made using post-inoculation medium. Five wells were inoculated with 100 μl at each dilution, plates were incubated at 37°C with 5% CO_2_ for 1 h, and then 100 μl post-inoculation medium was added. Plates were incubated at 37°C with 5% CO_2_ for 5 days, after which wells were subjected to IFA staining and evaluated for the presence of virus. Wells with specific staining were classified PEDV-positive. Based on the titration results, the 50% endpoint was calculated as 1 x 10^5^ TCID_50_/ml using the Reed-Muench method [30].

### Animals and animal care

The experiment was conducted in the Iowa State University Livestock Infectious Disease Isolation Facility (LIDIF), a biosafety level 2 research facility accredited by the Association for Assessment and Accreditation of Laboratory Animal Care (AAALAC). The facility was equipped with a single-pass non-recirculating ventilation system that provides directional flow from low contamination areas to high contamination areas and zones of negative pressure to prevent control airborne contamination from area-to-area or room-to-room. Each room was ventilated separately and humidity and temperature was strictly controlled.

Seven clinically healthy pregnant sows were acquired from one commercial sow farm at day 110 of their second gestation. To verify their negative status, sow fecal swabs were tested for PEDV, TGEV, and PDCoV using agent-specific rRT-PCRs and serum samples were tested for PEDV antibody using IFA.

Sows were housed in Danish-style free stall farrowing crates (Thorp Equipment Inc., Thorp, WI) and supplemental heat was provided for piglets. Animals were closely observed from the time they entered LIDIF to the end of the observation period by researchers, animal caretakers, and veterinary staff. All sows had been bred on the same day. To induce parturition, all sows were administered 10 mg of dinoprost tromethamine (Lutalyse^®^, Zoetis Inc., Florham Park, NJ) 24 h prior to the expected farrowing date, i.e., day 113 of gestation. Sows completed farrowing either 1 (n = 1), 2 (n = 5), or 4 (n = 1) days after induction. All viable piglets (n = 74) were ear-tagged and administered 1 ml iron hydrogenated dextran (VetOne^®^, Boise, ID) and 5 mg (0.1 ml) ceftiofur sodium (Excenel^®^, Zoetis). Piglets remained with their dam continuously throughout the 2-week observation period.

### Implementation of the experiment

#### Concentrated PEDV antibody

The procedure used to precipitate swine serum proteins and antibodies was a modification based on previous publications [31–32]. Specifically, swine serum proteins and serum antibody (IgG and IgA) were precipitated by single fractional precipitation using 30% and then 40% ammonium sulfate, respectively. The entire process was conducted in an environmental chamber (Caron^®^, Marietta, OH) maintained at 4°C. Initially, 2 PEDV naturally (field) exposed sows were acquired from a commercial swine farm. Testing of the pooled sow serum, before antibody purification and concentration, by PEDV WV ELISA resulted in S/P ratios of 5.0 for IgA and 2.6 for IgG. The 2 sows were humanely euthanized and exsanguinated to collect whole blood (ISU #2-14-7736-S). Serum was harvested, and stored in 1 L bottles (Biotainer^™^, Nalge Nunc Corp., Rochester, NY) at -20°C. For the first precipitation, the serum was thawed for 24 h at 4°C, the volume of saturated ammonium sulfate (Sigma-Aldrich) calculated to achieve 30% concentration was added in a drop-by-drop fashion while stirring continuously, and then the mixture was incubated at 4°C for 16 h with continued stirring. Following incubation, the mixture was centrifuged (4°C) at 4,000 x g for 10 min to remove protein aggregates and less soluble proteins. For the second precipitation, the volume of the supernatant (IgM-free antibody fraction) was measured, additional saturated ammonium sulfate was added as before to achieve a final concentration of 40%, and then the mixture was incubated for 16 h at 4°C with stirring. Thereafter, the mixture was centrifuged (4°C) at 4,000 x g for 10 min to recover the antibody fraction. The pelleted antibody was gently resuspended with PBS (1X pH 7.4) at a 1:5 (pellet:PBS) volume ratio. To remove salts, the solution was dialyzed in 250 ml dialysis flasks (Pierce^®^, Thermo-Fisher Scientific, Waltham, CA) floating vertically in ~15 liters of 4°C, continuously stirred PBS (1X pH 7.4). The entire volume of PBS was replaced every 4 h for 5 times and then the antibody solution was concentrated by polyacrylamide gel dialysis (Spectra/Gel^®^ Absorbent, Spectrum Laboratories, Inc. Rancho Dominguez, CA). The PEDV WV ELISA S/P ratios of the concentrated antibody solution were 3.0 for IgG, and 7.2 for IgA. The concentrated antibody solution was then aliquoted into 50 ml centrifuge tubes and stored at -80°C until used.

#### Treatments

The concentrated PEDV antibody solution was thawed at 22°C for 2 h and then 2-fold diluted with PBS (1X pH 7.4) to create 6 treatments, i.e., 5 dilutions (1:80; 1:160; 1:320; 1:640; 1:1280) of the antibody solution plus an antibody-negative control (PBS 1X pH 7.4). To assign treatments (Table 1), piglets were blocked by sow and randomized to treatments in a randomized block design using statistical software R (R 3.2.0, R foundation). Notably, all piglets remained with their dam, but all treatments were represented within each litter. Treatments were administered by intraperitoneal inoculation at the rate of 1.35 ml of the solution per kg of piglet bodyweight.

**Table 1:** Allocation of piglets to treatments by litter^a^.

Litter	Age (days) at time of treatment	No. of piglets	Groups (no. piglets within treatment)^b^					
			1^b^	2	3	4	5	6
1	5	13	2	3	2	2	2	2
2	4	11	2	1	2	2	2	2
3	4	9	1	2	1	2	2	1
4	4	10	2	1	2	1	1	3
5	4	10	2	2	1	2	2	1
6	4	9	2	2	2	1	1	1
7^c^	2	12	1	1	3	3	2	2
Totals		74	12	12	13	13	12	12

^a^ Piglets were assigned to treatment using randomized block design whereby all treatments were assigned to each litter.

^b^ Treatment 1 piglets served as negative controls. Piglets in treatments 2 to 6 were administered increasing levels of antibody (see Table 2).

^c^ Litter 7 was excluded from the study because the sow was agalactic.

#### PEDV inoculation

A virulent U.S. PEDV isolate (USA/IN19338/2013) was used as the challenge virus in this study. Isolation, propagation and characterization of this isolate was previously described [10]. On DPI 0, the PEDV stock solution (passage 7 in cell culture, 1 x 10^5^ TCID_50_/ml) was diluted to an estimated concentration of 1 x 10^3^ TCID_50_/ml, mixed 1:4 with milk replacer (Esbilac^®^, PetAg Inc., Hampshire, IL) and administered orally (5 ml) to each piglet. Thereafter, sows were monitored daily for diarrhea, milking ability, anorexia, and alertness. Piglets were monitored daily for diarrhea, rectal body temperature, dehydration, and ability to stand, walk, and suckle. Animals unable to suckle, reluctant to stand, or demonstrating ≥ 10% dehydration based on skin tenting were euthanized by intravenous administration of pentobarbital sodium (Fatal-Plus^®^, Vortech Pharmaceuticals, MI) at a dose of 100 mg/kg.

### Biological sample collection

#### Serum

Serum samples for antibody testing were collected from sows (DPIs -7, 14) and piglets (DPIs -4, 0, 14). Blood samples were drawn from the jugular vein or cranial vena cava using a single-use blood collection system (Becton Dickson, Franklin Lakes, NJ) and serum separation tubes (Kendall, Mansfield, MA). Blood samples were processed by centrifugation at 1,500 x g for 15 min, aliquoted into 2 ml cryogenic tubes (BD Falcon^™^, Franklin Lakes, NJ), and stored at -20°C until tested.

#### Mammary secretions

Colostrum and milk samples for antibody testing were collected from sows daily between DPIs -3 to 14. Sows were administered 20 USP units of oxytocin (VetOne^®^) to facilitate collection of mammary secretions. Samples were processed by centrifugation at 13,000 x g for 15 min at 4°C to remove fat and debris. The defatted samples were then aliquoted into 2 ml cryogenic tubes (BD Falcon^™^) and stored at -20°C until tested.

#### Fecal samples

Fecal samples for porcine coronavirus RT-PCR testing included fecal swab (BD BBL^™^ CultureSwab^™^ Collection/Transport system, Thermo-Fisher Scientific) samples collected from individual sows immediately prior to receipt of the animals and individual piglet fecal samples collected between DPIs 0 and 14. Approximately 1 gram of feces was collected from each piglet using a disposable fecal loop (VetOne^®^), mixed with 1 ml PBS (1X pH 7.4, Sigma-Aldrich) immediately after collection, placed in a 2 ml cryogenic tube (BD Falcon^™^), and stored at -80°C.

### Coronavirus reverse-transcriptase polymerase chain reactions (rRT-PCR)

#### RNA extraction

In brief, viral RNA was extracted from 100 μl of fecal swab samples and eluted into 90 μl of elution buffer using the Ambion^®^ MagMAX^™^ viral RNA isolation kit (Life Technologies) and a KingFisher^®^ 96 magnetic particle processor (Thermo-Fisher Scientific) following the procedures provided by the manufacturers.

#### Coronavirus primers and probes

Sow fecal swab samples and piglet fecal samples were tested for PEDV using a PEDV nucleocapsid (N) gene-based rRT-PCR described in Madson et al. (2014) and performed routinely at the Iowa State University-Veterinary Diagnostic Laboratory (ISU-VDL SOP 9.5263) [15]. Primers and probes targeting conserved regions of the PEDV N gene were designed to match a U.S. PEDV nucleotide sequences published in GenBank^®^ (accession no. KF272920) [15].

Sow fecal swab samples were tested for TGEV using a spike (S) gene-based rRT-PCR described in Kim et al. (2007) and performed routinely at the ISU-VDL (ISU-VDL SOP 9.5575). Primers and probes targeting conserved regions of the TGEV S gene were designed to match 9 TGEV strains, including Purdue 46-MAD (GenBank^®^ NC00236), TO14 (GenBank^®^ AF302264), TS (GenBank^®^ DQ201447), SC-Y (GenBank^®^ DQ443743), Miller M6 (GenBank^®^ DQ811785), TH-98 (GenBank^®^ AY676604), HN2002 (GenBank^®^ AY587884), 96–1993 (GenBank^®^ AF104420), and FS772/70 (GenBank^®^ Y00542) [33].

Sow fecal swab samples were tested for PDCoV using a membrane (M) gene-based rRT-PCR described in Chen et al. (2015) and performed routinely at the ISU-VDL (ISU-VDL SOP 9.5478) [34]. The protocol included positive control standards of known infectivity titers (TCID_50_). In brief, the forward primer, reverse primer, and probe were designed to match the M gene of global and U.S. PDCoV isolates. The probe was labeled with FAM/ZEN/3’Iowa Black Detector (Integrated DNA Technologies, Coralville, IA).

#### Real-time RT-PCR

The eluted RNA, primers, and probe were mixed with commercial reagents (Path-ID^®^ Multiplex One-Step RT-PCR kit, Life Technologies) and the RT-PCR reactions were conducted on an ABI 7500 Fast instrument (Life Technologies) as follows: 48°C for 10 min, 95°C for 10 min, 95°C for 15 s (45 cycles) and 60°C for 45 s. The real-time RT-PCR (rRT-PCR) results were analyzed using an automatic baseline setting with a threshold at 0.1. Quantification cycle (Cq) values ≤ 35 were considered positive for the corresponding coronavirus. Data were reported as "adjusted Cq":

Adjusted Cq=(35−sample Cq) (1)

### Coronavirus antibody assays

#### PEDV indirect immunofluorescence assay (IFA)

IFA plates were prepared by inoculating confluent monolayers of Vero cells (ATCC^®^ CCL-81^™^) in 96-well plates (CoStar^™^, Corning^®^) with 100 μl/well of PEDV (USA/IN19338/2013) at 1 x 10^3^ plaque-forming units/ml. The plates were then incubated for 18 to 24 h, after which the inoculum was removed and the cell monolayers fixed with cold acetone:alcohol (70:30) solution (Sigma-Aldrich). Plates were then air-dried, sealed, and stored at -20°C. To perform the test, serum samples were serially two-fold diluted (1:40 to 1:320) in PBS (1X pH 7.4) and then 100 μl of each dilution was transferred to IFA plates and incubated at 37°C for 1 h. After incubation, the diluted serum samples were removed from test plates, the plates rinsed 3 times with PBS (1X pH 7.4) and 50 μl of 1:50 diluted with fluorescein isothiocyanate (FITC) labeled mouse monoclonal antibody (Kirkegaard and Perry Laboratories, Gaithersburg, MD) was added to each well. After a 30 min incubation at 37°C, the plates were rinsed again with PBS (1X pH 7.4) and the cells were observed under an inverted fluorescent microscope for PEDV-specific cytoplasmic staining.

#### PEDV whole virus antibody ELISA

PEDV (USA/NC35140/2013) was used in the PEDV whole-virus based antibody ELISA. In brief, virus was propagated on Vero cells, the flasks subjected to one freeze-thaw, and the harvested material centrifuged at 4,000 x g for 15 min to remove cell debris. The virus was then pelleted by ultracentrifugation at 140,992 x g for 3 h, after which the virus pellet was washed twice with sterile PBS (1X pH 7.4). The purified virus was resuspended in PBS (1X pH 7.4) at a dilution of 1:100 of the original supernatant volume and stored at -80°C. Following titration and optimal dilution, polystyrene 96-well microtitration plates (Nalge Nunc Corp.) were manually coated (100 μl per well) with the viral antigen solution and incubated at 4°C overnight in a closed container containing a towel saturated with water. After incubation, plates were washed 5 times, blocked with 300 μl per well of a blocking solution containing 1% bovine serum albumin (Jackson ImmunoResearch Inc., West Grove, PA), and incubated at 25°C for 2 h. Plates were then dried at 37°C for 4 h and stored at 4°C in a sealed bag with desiccant packs. Plate lots with a coefficient of variation ≥ 10% were rejected.

ELISA conditions for the detection of anti-PEDV IgA and IgG antibodies in serum and colostrum/milk (defatted) specimens, including coating and blocking conditions, reagent concentrations, incubation times, and buffers, were identical. High positive, low positive, and negative plate controls, i.e., antibody-positive and -negative experimental serum or milk samples, were run in duplicate on each ELISA plate. All samples were diluted 1:50, after which plates were loaded with 100 μl of the diluted sample per well. Plates were incubated at 25°C for 1 h and then washed 5 times with PBST wash solution (PBS 1X, 0.1% Tween-20, pH 7.4).

To perform the assay, 100 μl of peroxidase-conjugated goat anti-pig IgG (Fc) antibody (Bethyl Laboratories Inc., Montgomery, TX) diluted 1:20,000 for serum and colostrum/milk samples or goat anti-pig IgA (Bethyl Laboratories Inc.) diluted 1:3,000 for serum and 1:45,000 for colostrum/milk samples was added to each well and the plates incubated at 25°C for 1 h. After a washing step, the reaction was visualized by adding 100 μl of tetramethylbenzidine-hydrogen peroxide (TMB, Dako North America, Inc., Carpinteria, CA) substrate solution to each well. After 5 min incubation at room temperature, the reaction was stopped by the addition of 50 μl of stop solution (1 M sulfuric acid) to each well. Reactions were measured as optical density (OD) at 450 nm using an ELISA plate reader (Biotek^®^ Instruments Inc., Winooski, VT) operated with commercial software (GEN5^™^, Biotek^®^ Instruments Inc.). The antibody response in serum and colostrum/milk samples was represented as sample-to-positive (S/P) ratios:

S/P ratio=(sample OD−blank well control mean OD/(positive control mean OD−blank well control mean OD) (2)

For serum, S/P ratios ≥ 0.80 were considered positive for PEDV IgG antibody [35].

#### PEDV fluorescent focus neutralization (FFN) assay

Colostrum, milk, and serum samples were tested for neutralizing antibody. Prior to FFN testing, defatted milk and colostrum samples were treated with Rennet (Rennet from Mucor miehei, Sigma-Aldrich). In brief, 5 μl Rennet was added to 1 ml of defatted milk or colostrum and briefly vortexed. The mixture was then incubated at 37°C for 30 min, vortexed, and then centrifuged at 2,000 x g for 15 min. The supernatant was then harvested and tested for neutralizing antibody.

To perform the FFN, test samples, antibody-positive control serum, and antibody-negative control serum were heat inactivated at 56°C for 30 min and then 2-fold serially diluted (1:4 to 1:512) in 96-well dilution plates (Axygen^®^, Corning^®^) using post-inoculation medium to give a final volume of 100 μl. Then, 75 μl of each dilution was transferred to new dilution plate (Axygen^®^, Corning^®^), mixed with 75 μl of PEDV (1 x 10^3.6^ TCID_50_/ml) to give final serum dilutions of 1:8 to 1:1024, and incubated at 37°C with 5% CO_2_ for 1 h. Vero cell confluent monolayers in 96-well plates (CoStar^™^, Corning^®^) were washed twice with post-inoculation medium, inoculated with 100 μl of the sample-virus mixture, incubated at 37°C with 5% CO_2_ for 1 h, and washed twice. 100 μl of post-inoculation medium was then added to each well and the plates incubated at 37°C with 5% CO_2_ for 48 h. Finally, cells were fixed with 80% cold acetone:alcohol (80:20), stained with FITC-conjugated monoclonal antibody (SD6-29, Medgene Labs, Brooking, SD) for 1 h, and observed under an inverted fluorescent microscope for PEDV-specific cytoplasmic staining. Positive neutralizing endpoints were determined as the highest dilution resulting in a ≥ 90% visual reduction in fluorescing foci relative to the antibody-negative serum control. Plates in which the positive control deviated more than 2-fold from its expected antibody titer were considered invalid.

### Analysis

Statistical analyses were performed using SAS^®^ 9.4 (SAS^®^ Institute Inc., Cary NC, USA). Body weight, percent change in body weight, body temperature, and fecal shedding (PEDV rRT-PCR Cq) were analyzed using linear mixed models. Treatment, DPI, and their interaction were analyzed as fixed effects and piglet and sow were analyzed as random effects. The time to death was analyzed using proportional hazard regression analyses, with a robust sandwich covariance matrix estimate to account for the dependence within the same sow. When no statistical significance was shown in the 6 group model, a 2 group (antibody-positive vs. antibody-negative) model was run. Percent change in body weight for any DPI was calculated relative to DPI -4:

Percent change=(weight−weight DPI−4)×100 (3)

Normal body temperature was defined as values within the 95% confidence interval calculated for the body temperature data collected on DPIs -4 and -1. The qualitative effect of PEDV infection body temperature was assessed by analyzing the proportion of piglets within the “normal range” of body temperature over time post inoculation using one-way ANOVA. The number of animals with normal body temperature (yes/no) and PEDV fecal shedding (yes/no) of each group were compared using one-way ANOVA.

Differences among sows in the number of surviving piglets was compared using the Kruskal-Wallis test. Differences among treatments in the time to death were analyzed using proportional hazard regression analysis with a robust sandwich covariance matrix estimate to account for dependence within the same sow.

## Results

### Sows

Sow fecal samples collected prior to inoculation were rRT-PCR-negative for PEDV, TGEV, and PDCoV. Likewise, serum samples collected from sows at DPI -7 were antibody-negative by IFA (<1:8) and PEDV WV ELISA (S/P <0.80). On this basis, all sows were considered PEDV-naïve at the time the experiment commenced.

The 7 sows farrowed a total of 74 liveborn pigs, 3 stillborn pigs, and 4 mummified fetuses (Table 1). Each piglet litter was kept intact and remained with its dam throughout the study. One sow and her litter were eliminated from the study on DPI 6 because the sow was agalactic. The remaining 6 sows were clinically normal throughout the study. No significant difference in number of survival piglets was detected among the 6 litters (Kruskal-Wallis test).

**Fig 1:**
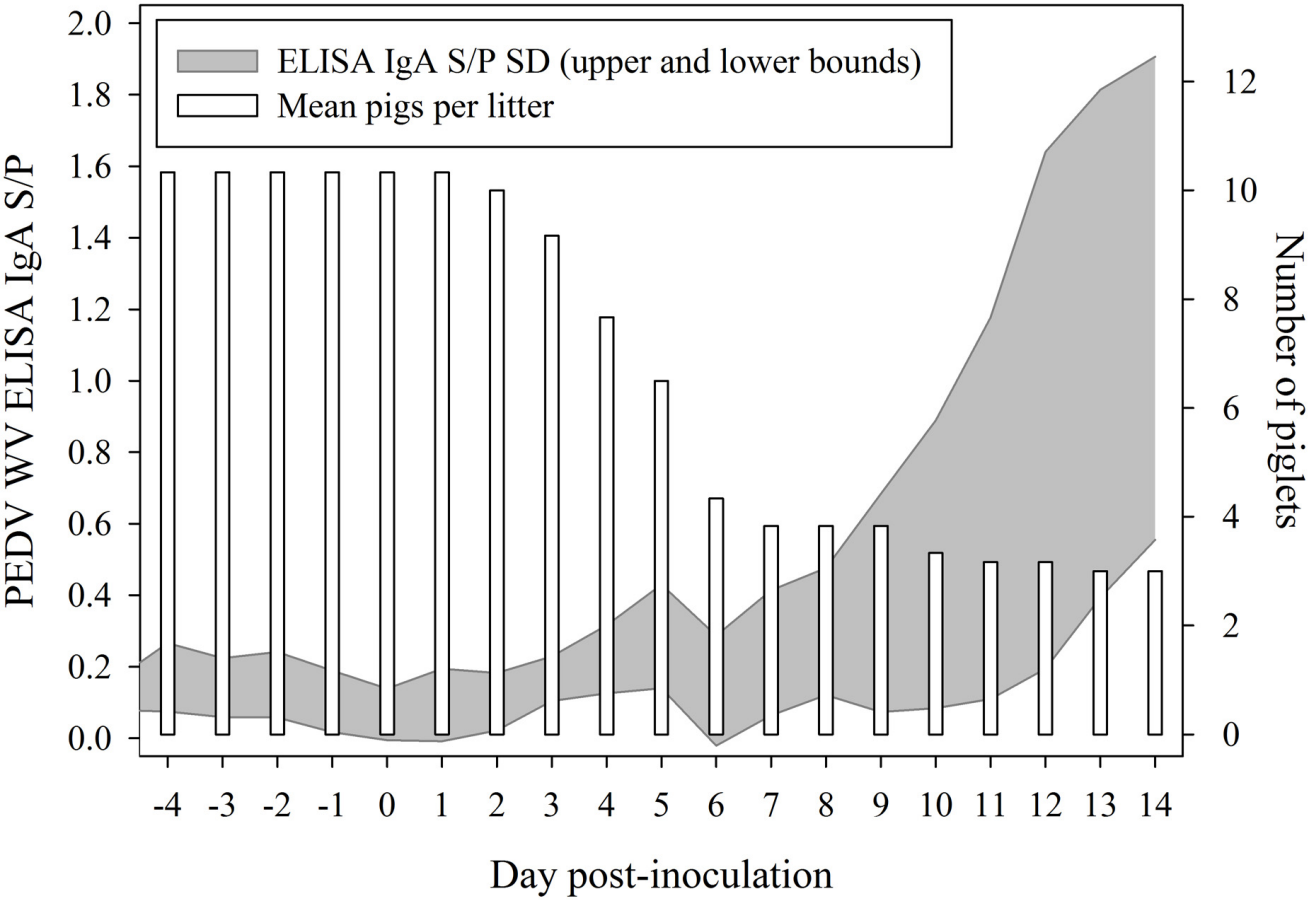
Anti-PEDV IgA in milk (standard deviation upper and lower bounds) based on daily samplings and mean number of piglets per litter (n = 6) over time post inoculation. No significant difference in survival was detected among the 6 litters (Kruskal-Wallis test).

Sow serum samples collected on DPI -7 had PEDV WV IgG ELISA S/P ratios between 0.2 and 0.6. By DPI 14, S/P ratios ranged from 1.2 to 2.7. The PEDV WV ELISA S/P ratios estimated for IgG and IgA in colostrum (within 48 h post-partum) were 0.97 and 0.16, respectively. Sow milk samples collected on DPI -5 had PEDV WV IgA ELISA S/P ratios between 0.0 and 0.2. By DPI 14, milk S/P ratios ranged between 0.4 and 2.0 (Fig 1). Rising anti-PEDV antibody levels in serum and milk indicate that the sows were infected with PEDV over the course of the experiment, presumably by exposure to PEDV-contaminated piglet feces.

### Piglets

Prior to PEDV inoculation, all piglets were clinically normal in appearance and behavior. Piglets were confirmed free of PEDV infection prior to inoculation on the basis of negative rRT-PCR results on individual piglet fecal samples collected on DPI 0.

**Fig 2:**
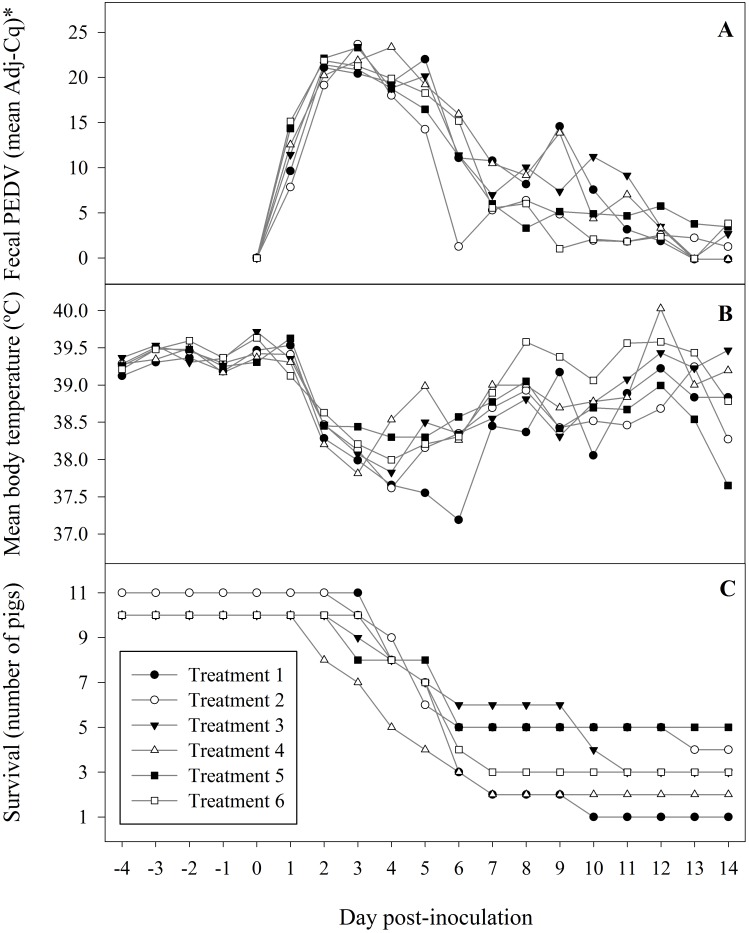
Treatment responses following inoculation of piglets with porcine epidemic diarrhea virus (USA/IN/2013/19338E). Treatment 1 piglets served as antibody-negative controls; piglets in treatments 2 to 6 had increasing levels of circulating anti-PEDV antibody. aAdjusted PEDV rRT-PCR quantification cycle (Cq) = (35 –sample Cq).

All piglets had normal feces on the day of inoculation, but 29% (n = 16), 69% (n = 43), and 89% (n = 50) of piglets had semi-solid or watery feces on DPIs 1, 2, and 3, respectively. A gradual resolution in the diarrhea was observed thereafter. Fecal loop samples from ≥ 1 pigs in each treatment group were PEDV rRT-PCR positive on DPI 1, with all samples positive on DPI 2. The highest concentration of virus in feces was observed between DPI 2 to 4 (Fig 2a). All fecal samples from piglets in the antibody-negative control group were positive through DPI 12. Fecal samples from the single remaining piglet in this group were negative on DPIs 13 and 14. PEDV rRT-PCR negative fecal samples were observed in the antibody-positive groups beginning on DPI 6, but positive fecal samples were recovered from 40% (n = 6) of antibody-positive pigs on DPI 14. Statistical analysis found no effect of treatment on the quantity of virus shed with treatment defined as 6 groups or defined as 2 groups (antibody-positive and antibody-negative control).

At the time of PEDV inoculation, mean piglet weight by treatment ranged from 1.9 to 2.3 kg. Piglets who survived to the end of the study gained 12.5 to150.0% of their body weight. No significant difference in the effect of PEDV on body weight was detected either with treatment defined as 6 groups (5 different PEDV antibody concentrations and an antibody-negative control) or with treatment defined as 2 groups (antibody-positive and antibody-negative control).

The quantitative effect of PEDV infection on body temperature over time is shown in Fig 2b. No significant difference in body temperature was detected with treatment defined as 6 groups, but a comparison based on 2 groups (antibody-positive vs antibody-negative) showed a significant difference in body temperature between the two groups on DPIs 4, 5, 6, and 8 (linear mixed model, p < 0.05).

The qualitative effect of PEDV infection body temperature was assessed by analyzing the proportion of piglets with "normal" body temperature (yes/no). Body temperature data collected on DPIs -4 to -1 showed a mean piglet body temperature of 39.3°C with a 95% confidence interval of 38.6°C to 40.1°C ("normal range"). All piglets were within the normal range on DPI 0. Piglets with body temperature below the lower bound of the normal range were observed from DPI 1 through 12. All survivor piglets had body temperatures within the normal range on DPIs 13 and 14. A statistical analysis of the body temperature data found no effect of treatment on the proportion of piglets with normal body temperature either with treatment defined as 6 groups or defined as 2 groups.

In total, 71% piglets (n = 44) died between 2 and 13 DPI (Fig 2c). Of these, 22.7% (n = 10) were humanely euthanized because they met previously established clinical criteria (unable to suckle, reluctant to stand, or ≥ 10% dehydration). Compared to the antibody-negative control group, a significant difference in time to death (treatment 5, p < 0.05), or a trend toward a difference (treatment 6, p = 0.11) was detected by hazard regression analysis.

As shown in Table 2, piglet serum samples collected on DPI -4 had FFN antibody titers <1:8, ELISA IgA S/P ratios ranging from 0.5 to 0.7, and ELISA IgG S/P ratios between 0.6 and 0.7 (IgG). Depending on the treatment, serum samples collected on DPI 0, i.e., 24 h post treatment, had FFN antibody titers ranging from <1:8 to 1:32 and ELISA S/P ratios of 0.2 to 3.3 (IgA) and 0.5 to 1.4 (IgG). Given the number of sampling points (DPIs -4, 0, 14) and the small sample size (number of surviving piglets) at the termination of the study, the PEDV antibody response on DPIs -4, 0, and 14 was analyzed using the Wilcoxon rank test. Using this approach, no significant differences in FFN, IgA, and IgG serum antibody responses were detected in treated versus control groups on DPIs -4 and 14, but most treatment groups had significantly higher antibody responses relative to antibody-negative piglets (treatment 1) on 0 DPI (Table 2).

**Table 2:** Serum antibody levels among treatment groups by day post inoculation.

Assay	Group	Day post inoculation		
		-4	0^a^	14
	1	<1:8 (4.6)	<1:8 (4.6)	1:64 (6.1)
	2	<1:8 (4.6)	1:5.3 (4.6)	1:19.7 (4.9)
FFN arithmetic mean (SE)	3	<1:8 (4.6)	1:6.1 (4.6)^b^	1:19.7 (4.9)
	4	<1:8 (4.6)	1:8.0 (4.6)^b^	1:32.0 (5.3)
	5	<1:8 (4.6)	1:17.1 (4.6)^b^	1:11.3 (4.9)
	6	<1:8 (4.6)	1:32.0 (4.6)^b^	1:16.0 (5.3)
	1	0.5 (0.2)	0.2 (0.2)	2.2 (0.4)
	2	0.7 (0.2)	0.7 (0.2)	2.0 (0.2)
PEDV IgA ELISA least square mean S/P (SE)	3	0.6 (0.2)	1.1 (0.2)	1.9 (0.3)
	4	0.6 (0.2)	1.8 (0.2)^b^	1.3 (0.3)
	5	0.6 (0.2)	2.8 (0.2)^b^	1.2 (0.2)
	6	0.7 (0.2)	3.3 (0.2)^b^	1.4 (0.3)
	1	0.6 (0.1)	0.5 (0.1)	1.7 (0.2)
	2	0.7 (0.1)	0.7 (0.1)^b^	1.0 (0.1)
PEDV IgG ELISA least square mean S/P (SE)	3	0.7 (0.1)	0.7 (0.1)^b^	1.5 (0.2)
	4	0.7 (0.1)	0.8 (0.1)^b^	1.3 (0.2)
	5	0.7 (0.1)	1.1 (0.1)^b^	1.0 (0.1)
	6	0.7 (0.1)	1.4 (0.1)^b^	0.9 (0.2)

^a^24 hours following intraperitoneal administration of concentrated PEDV antibody

^b^Significantly different from Group One (Wilcoxon rank test, p < 0.02)

## Discussion

The objective of this experiment was to expand our understanding of immunity against PEDV by quantifying the effect of circulating passive antibody on the course of PEDV infection in neonatal piglets using a "passive antibody model". Physiologically, this approach relied on the fact that antibodies injected into the peritoneum are quickly taken up by the lymphatic system and enter the circulatory system via the vena cava [36]. Essentially, intraperitoneal injection gives the same bioavailability as intravenous injection. Thus, it was possible to produce different levels of circulating PEDV antibody by intraperitoneal inoculation with specific levels of concentrated PEDV antibody solution (Table 2).

The passive antibody model has previously been used to study maternally-derived humoral immunity in mice [37–39], rats [40], and hamsters [41]. In swine, this method was previously used to study the effect of passive antibody on rotavirus infection [42–45] and porcine reproductive and respiratory syndrome virus [46–47]. Hodgins et al. (1999) found that passive antibody provided protection against clinical rotaviral infection, but also suppressed the piglets’ active immune responses [42]. Nguyen et al. (2006) reported that passive antibody protected neonates against rotavirus and determined that high titers of maternal antibody suppressed effector and memory B-cell responses [43]. Other workers found that intraperitoneal injection of PRRSV antibody sufficient to achieve serum neutralizing antibody titers of ≥ 1:16 inhibited PRRSV replication in 2 -to 5-week-old pigs [46–47].

In the current experiment, the effect of passive antibody on outcomes associated with PEDV infection, e.g., body weight, body temperature, survival, PEDV shedding in piglet feces, and serological responses to infection, was evaluated over a range of antibody treatment levels. The size of the experiment was limited by the number of piglets within treatments and the physical and logistical requirements of daily observations, sampling, and handling. The limitations of the experiment were offset by the experimental design. Specifically, the randomized block design allowed for random allocated of all treatments to all litters, thereby controlling for differences among sows.

In neonatal piglets inoculated with PEDV under experimental conditions, diarrhea typically occurs within one DPI [48–49], PEDV is detected in feces within 2 DPI [14, 15, 50], and mortality commences within 3 DPI [51]. A similar pattern was observed under the conditions of this experiment. Diarrhea was first observed at one DPI, all fecal samples were PEDV rRT-PCR positive on DPI 2, and mortality commenced on DPI 2. Statistical analyses found that circulating PEDV antibody did not protect piglets from the negative effects of PEDV on growth, reduce or eliminate shedding of PEDV in feces, or affect the humoral immune response against PEDV infection. However, circulating antibody partially ameliorated the effect of PEDV infection on body temperature and improved piglet survivability. Specifically, antibody-positive groups returned to normal body temperature faster (Fig 2b) and demonstrated higher survivability than PEDV antibody-negative control piglets (Fig 2c). These results are compatible with previous reports for swine coronaviruses. Shibata et al. (2001) reported that passive PEDV-specific antibody was effective in preventing PEDV infection and reduced mortality in 2 day-old piglets [51]. Stepanek et al. (1982) demonstrated that the presence of sufficient levels of TGEV-specific passive antibody delayed mortality due to TGEV infection in 4 day-old pigs [52]. The mechanisms responsible for producing these results have not been established, but we hypothesize that one or more of the following four mechanisms may be involved:

1. Although viremia was not confirmed in this experiment, a PEDV viremia lasting at least 7 DPI has been reported in young pigs inoculated under experimental conditions [18]. Since piglets administered concentrated antibody had FFN antibody titers of up to 1:32 at 0 DPI, it may be hypothesized that circulating neutralizing antibodies delivered via intraperitoneal administration may have reduced the level and/or the duration of PEDV viremia and modified the clinical course of the infection. There are no reports on PEDV with which these results can be compared, but the results would be consistent with a previous report by Lopez et al. (2007) showing that intraperitoneal administration of PRRSV-neutralizing antibody reduced or eliminated PRRSV viremia in young pigs (15 day-old) and delayed transmission to commingled sentinel pigs [47].
2. During PEDV viremia, binding of circulating PEDV antibodies (neutralizing and non-neutralizing) to viral antigenic determinants may have resulted in neutralization, agglutination, and/or complement fixation. This process could have facilitated the humoral and/or cellular immune responses by presenting antigen to the appropriate cells (dendritic cells, macrophages, and B cells) [53–55].
3. Antibody-dependent cell-mediated cytotoxicity (ADCC) effected by interactions between antibody and other components of the immune system, e.g. complement, phagocytic cells, and natural killer cells, could have expedited cell-mediated immune responses (CMI) against PEDV [56]. ADCC kills antibody-coated infected cells by inducing the expression of cell death-inducing molecules [57]. Presumably, this mechanism is effective for any PEDV-infected cells; not only enterocytes. Madson et al. (2015) has reported that, in addition to enterocytes, cells in the mesenteric lymph nodes and spleen may stain positive for PEDV by immunohistochemistry [17].
4. Passively-transferred, circulating PEDV IgG could have passed directly from capillaries into the small intestine by paracellular leakage and neonatal Fc receptors [42, 45, 58]. If so, the transported IgG may have neutralized PEDV in the intestinal lumen and/or assisted the humoral and CMI responses by facilitating uptake of PEDV antigen through receptors on apical surfaces of microfold cells [58–59]. Again, there is no PEDV research against which to test this hypothesis, but IgG is known to play an important role against parvovirus infection in crypt cells [60]. Evidence against this hypothesis is the fact that IgA is believed to play a primary role in protecting against enteric viruses that infect villous enterocytes, e.g. TGEV and rotavirus [61], and PEDV primarily infects villous enterocytes [17].

Combining the results of this experiment with previous work reported in the literature leads to the conclusion that both systemic antibody and maternal secretory IgA in milk contribute to the protection of the neonatal pig against PEDV infections. Beyond this observation lies a list of questions, e.g., What mechanism(s) effect protection? Which antibody isotype(s) and at what concentration are protective? How do the antibody levels and antibody isotypes examined in this experiment relate to those achieved in production settings by feedback, vaccination, or a combination of the two? Regardless, it is clear that the optimal protection to piglets will be provided by dams able to deliver both high antibody titers in colostrum and high titers of secretory IgA in milk.
